# Prevalence of fragile X‐associated tremor/ataxia syndrome: A survey of essential tremor patients with cerebellar signs or extrapyramidal signs

**DOI:** 10.1002/brb3.1337

**Published:** 2019-06-03

**Authors:** Ji‐Hyung Park, Wooyoung Jang, Jinyoung Youn, Chang‐Seok Ki, Byoung Joon Kim, Hee‐Tae Kim, Elan D. Louis, Jin Whan Cho

**Affiliations:** ^1^ Department of Neurology, Samsung Medical Center Sungkyunkwan University School of Medicine Seoul Republic of Korea; ^2^ Neuroscience Center, Samsung Medical Center Seoul Republic of Korea; ^3^ Department of Neurology Gangneung Asan Hospital, University of Ulsan College of Medicine Gangneung‐si Republic of Korea; ^4^ Department of Laboratory Medicine and Genetics Samsung Medical Center, Sungkyunkwan University School of Medicine Seoul Republic of Korea; ^5^ Department of Neurology, College of Medicine Hanyang University Seoul Republic of Korea; ^6^ Division of Movement Disorders, Department of Neurology Yale School of Medicine, Yale University New Haven Connecticut; ^7^ Department of Chronic Disease Epidemiology Yale School of Public Health, Yale University New Haven Connecticut; ^8^ Center for Neuroepidemiology and Clinical Neurological Research Yale School of Medicine, Yale University New Haven Connecticut

**Keywords:** cerebellar signs, essential tremor, extrapyramidal signs, *FMR1* premutation, fragile X‐associated tremor/ataxia syndrome

## Abstract

**Objectives:**

In screening studies of Western patients with cerebellar dysfunction, *FMR1* premutations have been detected. A screening study of East Asian patients with presumed essential tremor (ET) did not detect these mutations, possibly because the ET patients did not closely mimic the phenotype of fragile X‐associated tremor/ataxia syndrome (FXTAS). The aim of this study was to estimate the prevalence of *FMR1* premutations in a carefully recruited group of ET patients with additional phenotypic features of FXTAS.

**Materials and Methods:**

From April 2014 to April 2018, we prospectively recruited patients with ET diagnoses from three tertiary care centers. Demographic and clinical data were collected, as well as data on presence of cerebellar signs and extrapyramidal signs (EPS). Tremor, cerebellar signs, and EPS were evaluated using appropriate clinical rating scales. For ET patients with additional cerebellar signs or EPS, *FMR1* mutation analysis and brain magnetic resonance imaging were performed.

**Results:**

Six hundred and three ET patients were recruited. Cerebellar signs or EPS were present in 168 (27.9%) of 603. *FMR1* CGG repeat analysis was performed in 74 of 168 patients. Fifty‐two of 74 had cerebellar signs only, three had EPS only, and 19 had both neurologic abnormalities. Two patients had a *FMR1* premutation and fulfiled both clinical and radiological criteria of FXTAS.

**Conclusions:**

Two (2.7%) of 74 patients with presumed ET and additional neurological features were discovered to have FXTAS. The possibility of FXTAS should be considered in patients with ET who exhibit mild cerebellar signs or EPS.

## 1 INTRODUCTION

Fragile X‐associated tremor/ataxia syndrome (FXTAS) is a genetic disorder caused by an expanded CGG trinucleotide repeat located in the 5′ untranslated region of the fragile X mental retardation 1 (*FMR1*) gene. Fragile X syndrome results from a repeat number larger than 200 (i.e., the full mutation), while FXTAS results from a repeat number between 55 and 200, which is referred to as the premutation (Hagerman & Hagerman, 2013). The first cases of FXTAS were reported in 2001 (Hagerman et al., 2001). FXTAS primarily affects males, with a typical age of onset between 60 and 65 years (Hagerman & Hagerman, 2013). The main clinical features are slowly progressive intention tremor and gait ataxia. Other clinical features include peripheral sensory neuropathy, autonomic dysfunction, memory and executive function deficits, and extrapyramidal signs (EPS) (Hagerman & Hagerman, 2013). Various groups of movement disorder patients have been screened for FXTAS. As such, *FMR1* mutations have been detected in groups of Western patients whose main clinical feature was progressive cerebellar ataxia (Brussino et al., 2005; Macpherson, Waghorn, Hammans, & Jacobs, 2003; Van Esch et al., 2005). Similar studies performed in Western (Clark, Ye, Liu, & Louis, 2015; Deng, Le, & Jankovic, 2004; Garcia Arocena et al., 2004) or Eastern (Tan et al., 2004) essential tremor (ET) patients did not detect FXTAS. It is worthy of note, however, that the ET patients in those studies did not closely mimic the phenotype of FXTAS.

Essential tremor is a disorder with a wide spectrum of clinical features. Classically, ET has been considered a disorder with a benign long‐term course characterized by kinetic tremor without other neurological abnormalities. However, a growing number of studies now clearly demonstrate the coexistence of other neurological features. Patients with ET commonly exhibit cerebellar signs such as intention tremor or gait disorder (Arkadir & Louis, 2013; Deuschl, Wenzelburger, Loffler, Raethjen, & Stolze, 2000). Cognitive dysfunction (Lombardi, Woolston, Roberts, & Gross, 2001) or EPS (Thenganatt & Jankovic, 2016) have also been reported in ET patients. Of note, the postmortem examination of a woman with severe ET without parkinsonian features revealed Lewy bodies localized to the locus coeruleus, suggesting a connection between ET and Parkinson's disease (Louis et al., 2005). Currently, ET is considered a clinically heterogeneous disorder with numerous features of neurodegenerative disorders.

In fact, when ET is accompanied by such complex features (i.e., cerebellar signs, EPS), it can begin to resemble FXTAS. Indeed, patients with FXTAS have been assigned erroneous initial diagnoses of ET, before genetic analysis detected an *FMR1* premutation (Hall et al., 2005). These examples suggest that FXTAS is sometimes so similar to ET that it is difficult to distinguish the two entities on a clinical basis. To determine the prevalence of *FMR1* mutations in ET patients with cerebellar signs or EPS, we performed *FMR1* mutation analysis in ET patients who shared these clinical features of FXTAS.

## 2 MATERIALS AND METHODS

### 2.1 Subject selection

This research was approved by Samsung Medical Center Institutional Review Board and was conducted according to the principles expressed in the Declaration of Helsinki. We prospectively recruited consecutive ET patients who visited the movement disorder clinic at Samsung Medical Center, Hanyang University Medical Center, or Gangneung Asan Medical Center between April 2014 and April 2018. Informed consent was obtained from all individual participants included in the study. We adopted the criteria for “classic ET” from the Consensus Statement of the Movement Disorder Society in 1998 (Deuschl, Bain, & Brin, 1998), except for one of the exclusion criteria requiring absence of other abnormal neurologic signs. All enrolled patients had postural or kinetic tremor involving hands and forearms. Other neurologic abnormalities could be present, but they were only mild in all enrolled subjects and postural or kinetic tremor was the most prominent feature.

### 2.2 Clinical assessment and workup

Demographic data, age of tremor onset, presence of first‐degree relatives affected by tremor, and tremor response to alcohol were collected by history. Presence of rest, postural, or kinetic tremor, anatomic locations affected by tremor, and presence of cerebellar signs or EPS were determined by neurologic examination.

We excluded subjects who were confirmed with gene test to have autosomal dominant or recessive cerebellar ataxia syndromes other than FXTAS. We also excluded subjects who had bradykinesia and one or both of rest tremor and rigidity, which is the essential criterion of Parkinson's disease defined by the 2015 Movement Disorder Society criteria (Postuma et al., 2015). If EPS was progressive over time, those subjects were also excluded. In addition, we excluded subjects with prominent autonomic symptoms or signs and cerebellar sign or EPS suggestive of multiple system atrophy. We also excluded subjects with drug‐induced tremor, structural brain lesion or history of brain surgery, and severe medical problem that could produce tremor or gait disturbances, including endocrine disorders, hepatic disease, uremia, and severe joint problems.

Tremor was assessed by the revised version of the clinical rating scale for tremor (CRST) developed by Fahn and coworkers in 1993. Scale for the assessment and rating of ataxia (SARA) and Unified Parkinson's Disease Rating Scale (UPDRS) part 3 (Fahn & Elton, 1987) scores were also assigned to each patient. These scores were assigned by movement disorder neurologists (Jin Whan Cho and Jinyoung Youn).

**Figure 1:**
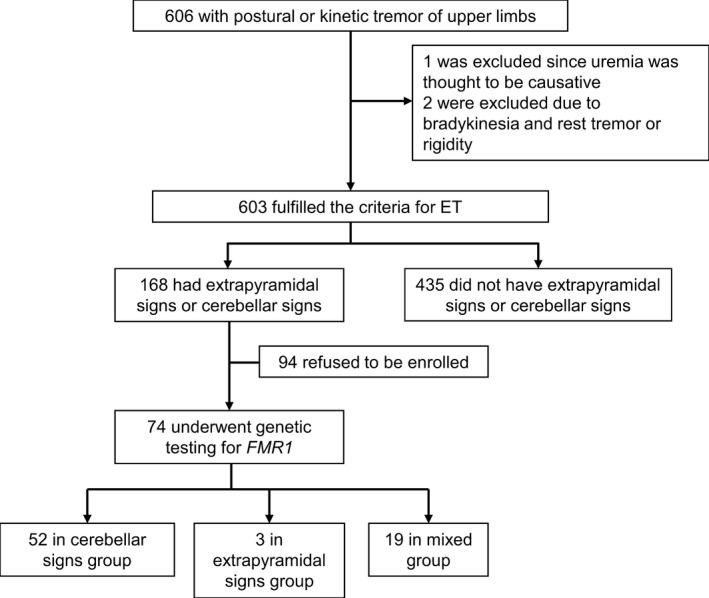
Flow diagram showing our patients enrollment and classification scheme

From the 603 recruited ET patients (Figure 1), we selected 168 patients who had cerebellar signs or EPS. Cerebellar sign was considered to be present when one or more of the following features was present: dysmetria or intention tremor during the finger‐to‐nose test, dysmetria during the heel‐to‐shin test, dysdiadochokinesia (hand) during the rapid alternating movements test, impaired tandem gait, and hypermetric saccades. EPS was considered to be present when at least one of the following signs was present: bradykinesia with decremental response, lead‐pipe rigidity, or tremor at rest. If the patient agreed, we performed *FMR1* CGG repeat number analysis and obtained a brain magnetic resonance image (MRI). The MRI was reviewed qualitatively for presence of middle cerebellar peduncle (MCP) or corpus callosum splenium (CCS) signs, both known to be useful diagnostic markers for FXTAS. All MRI scans were assessed by two experienced movement disorder neurologists, and a radiologic sign was thought to be present only when both agreed upon it.

### 2.3 Genetic analysis

The *FMR1* premutation screening was performed using a commercialized CGG repeat primed PCR (Asuragen Inc., Austin, TX). Testing was conducted based on the manufacturer's recommendations, as previously described (Chen et al., 2010). In this assay, full‐length and CGG repeat primed amplicons were produced using two flanking gene‐specific primers and one CGG repeat primer and were analyzed by capillary electrophoresis.

### 2.4 Statistical analysis

We compared ET patients with versus without additional features (cerebellar signs or EPS) using chi‐squared tests, Fisher's exact tests, or Student's *t* tests where appropriate (Table 1). For the presence of an affected first‐degree relative or alcohol response, possible answers were “yes”, “no”, or “unknown” and statistical analysis was performed based on a 2 × 3 contingency table.

**Table 1:** Demographic data and clinical characteristics of essential tremor patients

	Essential tremor without additional features (*n* = 435)	Essential tremor with additional features (*n* = 168)	*p*‐value
No of women (% in each group)	201 (46.2%)	49 (29.2%)	<0.001*
Age at onset, years (mean ± *SD*)	54.0 ± 18.1	56.83 ± 15.7	0.061
No of patients with affected first‐degree relative (% in each group)	135 (31.0%)	67 (39.9%)	0.112
No of patients with alcohol response (% in each group)	73 (16.8%)	39 (23.2%)	0.140
No of patients with unknown alcohol response (% in each group)	236 (54.3%)	79 (47.0%)	0.140
No of patients with head tremor (% in each group)	49 (11.3%)	31 (18.5%)	0.020*
No patients with voice tremor (% in each group)	28 (6.4%)	22 (13.1%)	0.008*
No of patients with leg tremor (% in each group)	2 (0.5%)	1 (0.6%)	1.000

Note

Pearson's chi‐squared test, Student's *t* test, or Two‐tailed Fisher's exact test was used.

Abbreviation: *SD*, standard deviation.

* Statistically significant.

In addition, we divided the subjects who underwent *FMR1* analysis into three groups: those with cerebellar signs and no EPS (“cerebellar signs group”), with EPS and no cerebellar signs (“EPS group”), and with both of cerebellar signs and EPS (“mixed group”). Demographic data, clinical data, CRST score, and presence of abnormal MRI signs were compared between all three groups using analysis or variance or Kruskal‐Wallis *H* test according to whether the data followed a normal distribution. SARA score was compared between EPS group and one of cerebellar signs group or mixed group. UPDRS part 3 score was compared between cerebellar dysfunction group and one of EPS group or mixed group. Student's *t* test or Mann‐Whitney *U* test was used for analysis between two groups according to whether data followed normal distribution. *p* values of <0.05 were considered statistically significant. Statistical analyses were performed using a commercially available software package (PASW version 23.0; SPSS Inc., Chicago, IL, USA).

## 3 RESULTS

A flow diagram describing our patient enrollment and classification strategy is represented in Figure 1. Six hundred and three patients fulfilled our criteria to be enrolled as ET. Among them, 168 patients had either cerebellar signs or EPS, and the remainder did not. Demographic data and clinical characteristics of each group are summarized in Table 1. The proportion of women was significantly higher among patients with ET without cerebellar signs or EPS (46.2% vs. 29.2%, *p* < 0.001) while voice tremor (13.1% vs. 6.4%, *p* = 0.008) and head tremor (18.5% vs. 11.3%, *p* = 0.020) on examination was more common in ET patients with cerebellar signs or EPS (Table 1). Among 74 patients who agreed to undergo *FMR1* mutation analysis, 52 were in the cerebellar signs group, three in the EPS group, and 19 in the mixed group. Table 2 summarizes demographic data, clinical rating scales, *FMR1* CGG repeat analysis, and brain MRI results in three groups. UPDRS part 3 score was significantly lower in cerebellar signs group compared to EPS group (*p* = 0.049) or mixed group (*p* < 0.001). Mean SARA score was higher in cerebellar signs group or mixed group compared to EPS group, and it was marginally significant (*p* = 0.092 for cerebellar vs. EPS group, *p* = 0.055 for mixed vs. EPS group). In all patients with positive CCS sign, hyperintensity was adjacent to the ventricle and spanned over less than half of thickness of the splenium of the corpus callosum.

**Table 2:** Characteristics of the patients who underwent *FMR1* CGG repeat analysis

	Cerebellar signs group (*n* = 52)	Extrapyramidal signs group (*n* = 3)	Mixed group (*n* = 19)	*p*‐value
No of women (% in each group)	5 (9.6%)	0 (0.0%)	1 (5.3%)	1.000^a^
Age at onset, years (mean ± *SD*)	56.4 ± 14.9	49.7 ± 25.5	60.1 ± 15.3	0.454^a^
No of patients with onset at age of 50 or later (% in each group)	39 (75.0%)	1 (33.3%)	15 (78.9%)	0.171^a^
Disease duration, years (mean ± *SD*)	11.8 ± 11.9	21.0 ± 24.6	6.4 ± 9.4	0.054^a^
CRST (mean ± *SD*, minimum, maximum)	26.4 ± 19.0, 3, 119	20.7 ± 19.3, 9, 43	23.7 ± 10.9, 12, 56	0.591^a^
SARA (mean ± *SD*, minimum, maximum)	4.0 ± 3.2, 0.5, 14	1.3 ± 2.3, 0, 4	5.8 ± 3.4, 1, 12.5	0.092^b^, 0.055^c^
UPDRS part 3 (mean ± *SD*, minimum, maximum)	6.4 ± 2.7, 1, 20	8.0 ± 0.0, 8, 8	12.5 ± 5.0, 6, 23	0.049^b^, *, <0.001^d^, *
No of patients with rigidity (% in each group)	0	0 (0.0%)	10 (52.6%)	0.221^c^
No of patients with rest tremor (% in each group)	0	3 (100.0%)	7 (36.8%)	0.078^c^
No of patients with bradykinesia (% in each group)	0	0 (0.0%)	3 (15.8%)	1.000^c^
No of patients with *FMR1* premutation (% in each group)	1 (1.9%)	0 (0%)	1 (5.3%)	0.509^a^
No of patients with MRI (% in each group)	21 (40.1%)	1 (33.3%)	12 (63.2%)	0.238^a^
No of patients with positive MCP sign (% among those who did MRI)	1 (4.8%)	0 (0%)	1 (8.3%)	1.000^a^
No of patients with positive CCS sign (% among those who did MRI)	7 (33.3%)	1 (100.0%)	5 (41.7%)	0.445^a^

Note

Two‐tailed Fisher's exact test, Kruskal‐Wallis H test, or Mann‐Whitney U test was used.

Abbreviations: CCS, corpus callosum splenium; CRST, clinical rating scale of tremor; MCP, middle cerebellar peduncle; MRI, magnetic resonance imaging; SARA, scale for assessment and rating of ataxia; *SD*, standard deviation; UPDRS, unified Parkinson's disease rating scale.

a Between all three groups.

b Between cerebellar signs group and extrapyramidal signs group.

c Between extrapyramidal signs group and mixed group.

d Between cerebellar signs group and mixed group.

* Statistically significant.

**Figure 2:**
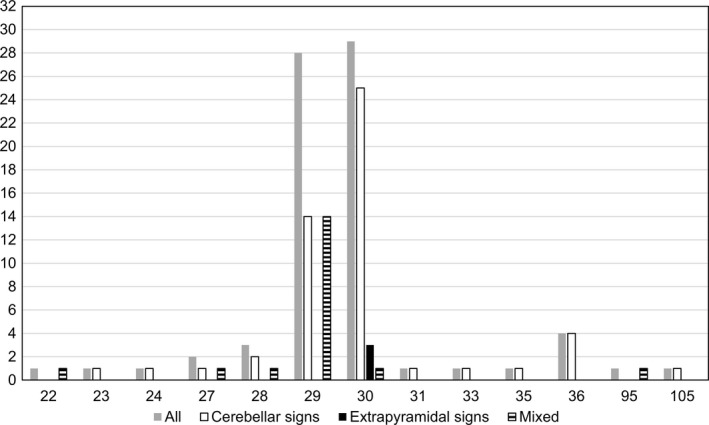
Distribution of *FMR1* CGG repeat numbers. Colors and patterns of the columns indicate patient groups, and height of the columns represent the number of subjects corresponding to each repeat number

The distribution of *FMR1* CGG repeat number is illustrated in Figure 2. The most common repeat numbers were 29 and 30 among all subjects (accounting for 37.8% and 39.2%, respectively). In the cerebellar signs group, these values were 26.9% and 48.1%. In the EPS group, they were 0% and 100%, and in the mixed group, 73.7% and 5.3%. Two patients, whom we denote as patient 1 and patient 2, had *FMR1* premutation. Patient 1 was from cerebellar signs group (repeat number = 105) and patient 2 was from mixed group (repeat number = 95).

**Figure 3:**
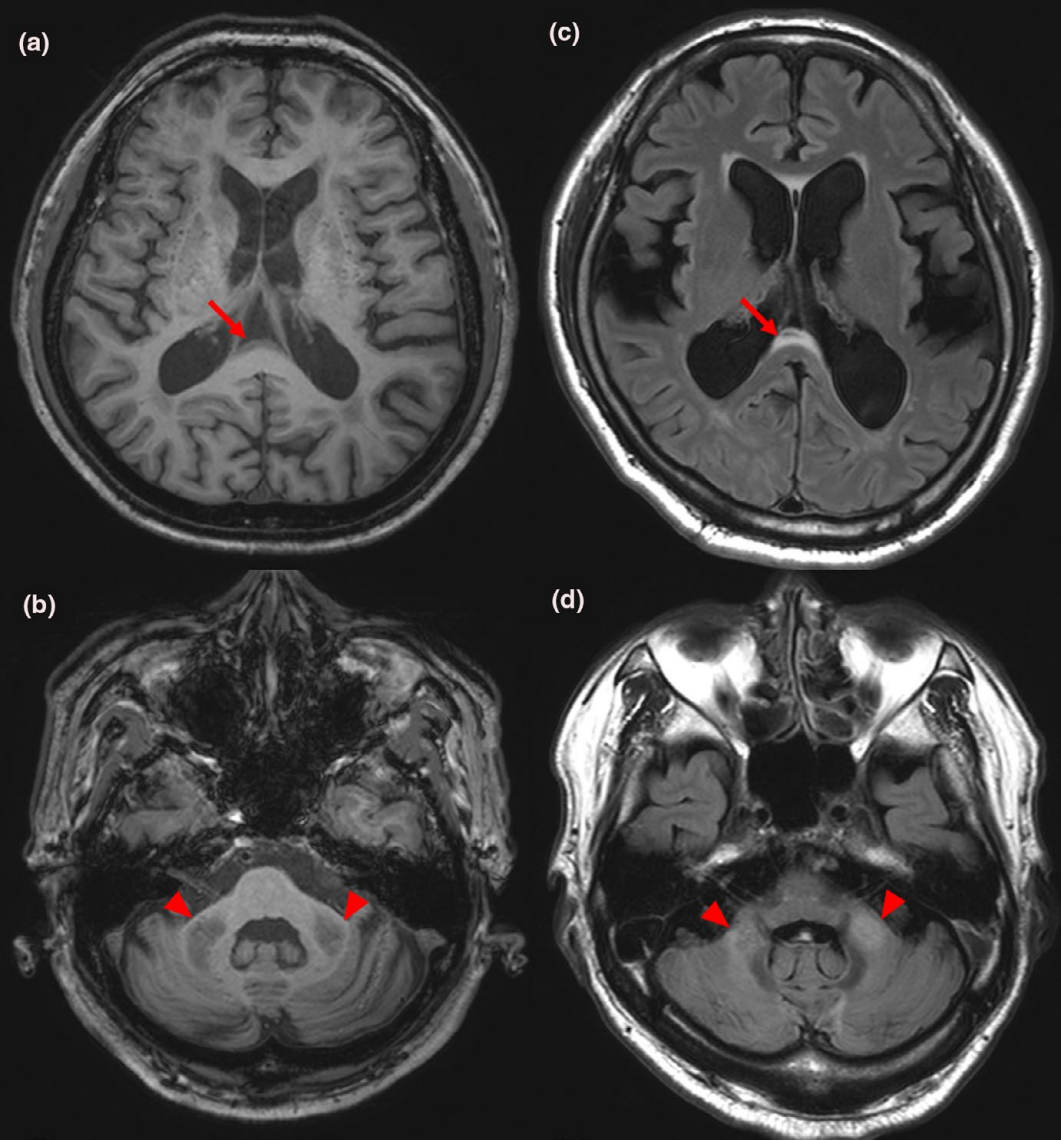
Magnetic resonance images of patients with *FMR1* premutation. Panels (a) and (b) are images of patient 1, and panels (c) and (d) are images of patient 2. Panels (a) and (c) shows corpus callosum splenium sign (red arrows), and panels (b) and (d) shows middle cerebellar peduncle sign (red arrowheads)

Both patients with *FMR1* premutation were revealed to have FXTAS. Patient 1 was a 59‐year‐old man at the time of the genetic study. He began to have tremor of both hands while reaching an object at age of 53. On his initial visit of our clinic at age of 55, neurologic examination was unremarkable except mild postural and kinetic tremor of both hands. At age of 58, however, he developed mild gait instability but was able to walk on himself. Neurologic examination revealed postural and kinetic tremor of similar intensity as before, mild dysmetria of both arms, and occasional mild truncal swaying with tandem walking. EPS were not observed. At this time, CRST score was 17, SARA score was 13, and UPDRS part 3 score was 20. Detailed neuropsychiatric test battery results were normal. Patient 2 was a 64‐year‐old man at the time of the genetic study. He developed bilateral hand tremor present with hand movement at age of 60. On examination done at age of 62, he had postural and kinetic tremor of both hands, minimal swaying during tandem walking but otherwise normal gait, and mild rigidity of the right arm without bradykinesia. Follow‐up examination done at age of 64 revealed more prominent swaying with tandem walking but intact straight walking, and mild limb dysmetria. Rigidity of the right arm was not changed. At age of 64, CRST score was 13, SARA score was 10, and UPDRS part 3 score was 18.5. Korean version of the Montreal Cognitive Assessment score was 25/30. In both patients, postural or kinetic tremor was the most prominent feature initially and diagnosis of ET could be made, but they later developed additional features. Brain MRI of both patients done at the time of genetic testing showed CCS sign and prominent MCP sign (Figure 3). CGG repeat number of *FMR1* gene was expanded to 105 and 95, respectively. Both patients fulfilled the major clinical and major radiological criteria and were diagnosed with definite FXTAS according to the criteria published in 2003 (Jacquemont et al., 2003).

## 4 DISCUSSION

This is the first study investigating the prevalence of FXTAS in a cohort of patients with ET and additional FXTAS‐like clinical features. As such, the clinical features of the ET cases in our study resembled those of FXTAS more closely than previous similar studies; in those studies, *FMR1* premutation was screened for in large groups of ET patients without further restricting phenotype (Clark et al., 2015; Deng et al., 2004; Garcia Arocena et al., 2004). In contrast, we recruited only ET patients with additional mild cerebellar signs or EPS, which are important clinical features of FXTAS. We found two cases of FXTAS from 74 patients (2.7%), which is a high proportion considering the low prevalence of *FMR1* premutation in Korea. Several population‐based studies in Korea investigated the prevalence of *FMR1* premutation carriers by utilizing data on women of reproductive age and estimated it to be between 1/788 and 1/1090 (Han et al., 2012; Jang et al., 2014). This is lower than previously reported prevalence of *FMR1* premutation carriers in Western countries. In the female population, it was 1/259 in a Canadian study (Rousseau, Rouillard, Morel, Khandjian, & Morgan, 1995). In the male population, the estimated prevalence ranged between 1/400 and 1/813 in Caucasians (Dombrowski et al., 2002; Hantash et al., 2011).

The prevalence of FXTAS in the screened patients of our study was 2.7% (2/74). This is comparable to the pooled prevalence of FXTAS in male cerebellar ataxia patients, which is 1.5% (16/1049), reported in a meta‐analysis of *FMR1* premutation screening studies (Jacquemont, Leehey, Hagerman, Beckett, & Hagerman, 2006). A guideline of FXTAS testing was proposed (Berry‐Kravis et al., 2007); indeed, it suggested that testing for *FMR1* mutation be considered if a patient developed action tremor with parkinsonism or cognitive decline after age of 50 years, which would make a clinician think of testing *FMR1* mutation in ET patients. However, previous *FMR1* screening studies in ET did not detect any cases of FXTAS. Our study provided a positive result of screening for *FMR1* premutation in patients whose main clinical picture was ET with additional mild cerebellar signs or EPS. It could help selecting future candidates for testing of *FMR1* premutation.

Middle cerebellar peduncle sign is a highly specific radiologic marker for FXTAS, with a reported sensitivity of 0.52 and specificity of 0.87 (Jacquemont et al., 2003). CCS sign is another imaging marker, defined by hyperintensity in the splenium of the corpus callosum. One study reported its sensitivity and specificity in men to be 0.87 and 0.6, respectively (Deborah, Hall, & P, Meghan Hermanson BS, Emily Dunn MD, The, 2016). In our study, MCP sign was present only in patients with FXTAS. Among subjects without MCP sign, no one was found to have *FMR1* premutation despite similar clinical and demographic characteristics, with a negative predictive value of 100%. This result suggests that in a patient with ET and additional cerebellar signs or EPS, FXTAS would be less likely without the MCP sign. Only one of six patients with positive CCS sign was diagnosed with FXTAS, with a positive predictive value of 16.7%. This suggests that CCS sign in an ET patient with additional cerebellar signs or EPS would not be a good predictor of FXTAS.

From our study subjects, the two most common *FMR1* CGG repeat numbers were 30 and 29. Patients corresponding to those two numbers accounted for most of our study subjects (77.0%). Thirty and twenty‐nine are also the most common repeat numbers in a larger sample from the Korean population and in populations from other countries (Jang et al., 2014; Peprah, 2012). Therefore, the genetic distribution with regard to *FMR1* in our study subjects likely represents the general population.

Mean of UPDRS part 3 score was significantly higher in the EPS or mixed group compared to the cerebellar signs group, which reflected our patient grouping scheme. However, mean of SARA score did not differ significantly between cerebellar signs or mixed group and EPS group. Nevertheless, the mean value was the smallest in the EPS group, and it was marginally significant. This lack of statistical significance may be due to the very small number of subjects in the EPS group. Moreover, kinetic tremor of upper limbs, which all of the subjects had, partly contributed to the SARA score and might blunted difference between groups.

Proportion of patients with voice tremor and patients with head tremor were significantly higher among ET patients with additional features compared to ET patients without additional features. A recent study reported abnormal cerebellar activity in ET patients with head tremor compared to those having ET without head tremor (Wang et al., 2018). In addition, presence of axonal torpedoes in vermis was described in ET patients with tremors of neck, voice, and jaw (Louis et al., 2011). Those results suggest that head and voice tremor is related to cerebellar dysfunction. Since ET patients with clinical signs of cerebellar dysfunction would have more severe involvement of the cerebellum, it is logical that head tremor and voice tremor is more common in patients having ET with additional signs. Indeed, most of patients in ET with additional signs group in our result had signs of cerebellar dysfunction. The abundance of male patients among ET patients with additional signs in our result is not fully explainable. To our best knowledge, different frequency of EPS or cerebellar dysfunction in ET patients by sex had not been reported. Sex hormones or genetic or epigenetic factors related to the sex chromosomes might have some influence on development of additional signs in ET. Possibility of selection bias could not be excluded.

Before our study, only one case of FXTAS had been reported in Korea (Ehm, Yang, Kim, & Jeon, 2014). The patient was a 75‐year‐old man who had developed resting tremor 6 years previously, which began in the right hand and progressed to the left hand. He also had mild terminal tremor in his left hand with subtle dysmetria, progressive gait ataxia, and major depressive disorder. MRI of the brain revealed bilateral MCP sign. Genetic analysis revealed an *FMR1* CGG repeat number of 136.

Our study suggests that if a patient presents with dominant clinical feature of ET but also has mild cerebellar sign or EPS, brain MRI should be done to exclude structural causes or genetic disorders, especially FXTAS. Positive MCP sign, which had a 100% positive predictive value in our study, would strongly predict FXTAS. Moreover, the possibility of FXTAS might be higher if a patient with apparent ET would have both of mild cerebellar sign and EPS, not only one of them.

The strength of this study is that we specified detailed inclusion criteria and recruited a patient group more closely resembling FXTAS patients than subjects of previous studies. Men comprised 91.9% of the patients who underwent *FMR1* mutation analysis, and age of onset was 50 years or older in 74.3% of them. This yielded a higher prevalence of FXTAS than previous studies with ET patients. In addition, we rated severity of tremor, cerebellar signs, and EPS by dedicated scales in every subject who underwent test for *FMR1*. This study also has several limitations. First, the number of patients who underwent *FMR1* mutation analysis was small because ET patients with cerebellar signs and/or EPS were not very frequent. Second, the study subjects may not be representative of entire ET patients because they were recruited only from tertiary care centers. Finally, family history of mental retardation suggestive of fragile X syndrome, which could be a clue for presence of *FMR1* premutation in the proband, was not investigated.

In summary, the result of our study implies that FXTAS might comprise a significant portion of patients with ET and additional cerebellar signs or EPS. One should suspect FXTAS and consider performing *FMR1* mutation analysis for an ET patient who also have cerebellar signs or EPS. This would help find new cases of FXTAS, especially in East Asia.

## CONFLICT OF INTEREST

The authors declare that they have no conflict of interest.

## Data Availability

The data that support the findings of this study are available on request from the corresponding author. The data are not publicly available due to privacy or ethical restrictions.
